# Significance of IL-6 Deficiency in Recognition Memory in Young Adult and Aged Mice

**DOI:** 10.1007/s10519-019-09959-6

**Published:** 2019-05-25

**Authors:** Izabela Bialuk, Piotr Jakubów, Maria Małgorzata Winnicka

**Affiliations:** Department of General and Experimental Pathology, Medical University of Białystok, Mickiewicza 2c, 15-222 Białystok, Poland

**Keywords:** IL-6 deficiency, Object recognition memory, Holeboard, Elevated plus maze, Mice

## Abstract

Chronic peripheral elevation of interleukin 6 (IL-6) in humans is associated with cognitive deficits. 4- and 24-month-old IL-6-deficient C57BL/6J (IL-6KO) and reference wild-type (WT) mice were tested in an object recognition test. Discrimination ratios and recognition indexes were significantly lower in 4-month-old IL-6KO and in 24-month-old WT mice vs 4-month-old WT animals. Their discrimination ratios had negative values and recognition indexes were below 50% indicating inability to differentiate the novel from the familiar object after 1-hour delay. In 24-month-old IL-6KO mice recognition index reached 53.17% indicating that their recognition memory was not worsened with age in comparison with younger IL-6-deficient animals. Results of holeboard and elevated plus maze indicated that this effect was memory specific. Inborn IL-6 deficiency attenuated recognition memory in 4-month-old mice and did not altered recognition memory in aged animals. IL-6 signalling may constitute a target for development of the protection against memory disturbances connected with IL-6 overexpression.

**Electronic supplementary material:**

The online version of this article (10.1007/s10519-019-09959-6) contains supplementary material, which is available to authorized users.

## Introduction

Interleukin 6 (IL-6) is a multifunctional cytokine, with context-dependent pro- and anti-inflammatory properties (Hunter and Jones 2015; Trapero and Cauli 2014; Yirmiya and Goshen 2011). Clinical observations on humans have demonstrated an age-associated chronic peripheral increase in IL-6 level inversely correlated with cognitive decline (Marsland et al. 2006; Mooijaart et al. 2013; Simpson et al. 2013; Weaver et al. 2002; Wright et al. 2006). Moreover, the detrimental role of IL-6 in age-related memory disturbances was demonstrated in senescence-accelerated mice (Tha et al. 2000) implying that IL-6 is involved in the impairment of learning and memory processes in normal aging (Godbout and Johnson 2004; Weaver et al. 2002; Wright et al. 2006).

Despite the facts pointing to the important role of IL-6 in neuroinflammatory and neurodegenerative diseases little is known about biological actions of this cytokine under physiological conditions and how they affect cognition (McAfoose and Baune 2009). It could be expected that because of a deleterious effect of increased IL-6 level on cognitive functions, its deficiency would be beneficial for these processes. However, in our previous study, 12–14-week-old, IL-6-deficient mice showed impaired memory processes in an object recognition test (Hryniewicz et al. 2007). This unexpected result concerning recognition memory was also described in a novel object recognition test performed on 6-month-old IL-6-deficient mice (Baier et al. 2009). Moreover, results of our study performed in Morris water maze (Bialuk et al. 2018), evaluating spatial memory, showed significant attenuation of learning ability in IL-6-deficient mice that was more pronounced in younger than in aged animals. However, it was difficult to answer the question whether inborn IL-6 deficiency slows down age-related memory decline because the swimming performance in IL-6 knock-out mice was significantly slower than in control, producing endogenous IL-6, animals. Therefore, in the current study we compared cognitive processes in 4- and 24-month-old IL-6-deficient and age-matched control mice in an object recognition test, less affected by locomotor activity alterations, and closely related to conditions under which human recognition memory is evaluated (Ennaceur and Delacour 1988).

## Materials and methods

### Animals

Naïve, male young adult (4-month-old) and aged (24-month-old) IL-6-deficient mice C57BL/6J^IL−6−/−^^TMKopf^ (IL-6KO) and reference age-matched wild-type (WT) animals (C57BL/6J) were used. Each group consisted of 10 mice. Animals, obtained from the Centre for Experimental Medicine of the Medical University of Białystok, originally purchased from the Jackson Laboratory (USA), were used in experiments performed after at least 14 days of acclimatization to the laboratory conditions. The mice were maintained in a temperature-controlled conditions (22 ± 1 °C) with a 12 h light–dark cycles beginning at 7 am and were housed in polycarbonate cages, five animals per cage, with water and commercial food available ad libitum. Before experiments mice were handled for cage cleaning and weighing. All experiments were approved by the Local Animal Ethics Committee in Bialystok, Poland and were performed in compliance with the European Communities Council Directive 2010/63/EU.

### Experimental design

Behavioral tests were carried out between 8.30 am and 12.30 pm in an air-conditioned, sound-isolated room with regulated light intensity. During one experiment five animals of both genotypes were submitted to three behavioral tests. On day 1 a holeboard test, followed by an elevated plus maze test were carried out. Subsequently, on the same day a habituation session in an object recognition apparatus was conducted. On day 2 an object recognition test was performed. Apparatuses were cleaned with 70% ethanol after each group of animals. An investigator, who was not familiar with the animal’s genotype and age, carried out experiments. Subsequently, an independent researcher who was given an evaluation form watched recorded experiments and assessed animal’s behaviour.

### Behavioral tests

#### Holeboard test

The experiments were performed according to the modified method previously described (File and Wardill 1975). The apparatus was a grey wooden box with a square floor of 53.5 cm × 53.5 cm divided into 25 equal parts and surrounded by a 42 cm high wall. Four holes in the floor (2.5 cm in diameter) were designed as objects of possible interest to the animals. The apparatus was placed on the floor and lit with the intensity of 30 lx. The animal was placed in the center of the holeboard box and its behavior was observed for 5 min. Locomotor activity (ambulation, horizontal activity) was measured as the number of squares crossed with all four limbs. Exploratory activity (vertical activity) was measured as the number of rearing events (rises of an animal on its rear limbs, either with forelegs leaning against the wall or away from the wall), and the number of head-dips (when a mouse lowered its head into a hole, so the eyes disappeared beneath the plane of the floor). During assessment of ambulation, horizontal and vertical activities were recorded. The number of crossed squares adjacent and not adjacent to the apparatus walls was used to measure peripheral and central activity, respectively. Moreover, latency time to leave the central area was also recorded to measure anxiety level.

#### Elevated plus maze

The procedure was performed immediately after the holeboard test, according to the modified method previously described (Pellow et al. 1985). The apparatus, made of the same material as the holeboard box, was raised 80 cm above the floor with constant illumination of 75 lx at its level. The elevated plus maze consisted of four arms: two open, 30 cm × 7 cm, and two closed arms 31 cm × 7 cm × 35 cm, with an open roof. The arms were arranged in such a manner that the two open arms were opposite to each other and connected with the central area 7 cm × 7 cm. Mice were placed in the central area of the maze, facing one of the open arms. The number of arm entries and the time spent in each type of the arm, as well as the time spent in the central area were counted for 5 min of observation.

#### Object recognition test

The procedure was performed according to the method previously described (Ennaceur et al. 1997; Ennaceur and Delacour 1988) and it may be summarized as follows. The apparatus was a gray wooden box (52.5 × 37.5 × 41.5 cm) placed on the floor and lit with the intensity of 40 lx in a sound isolated room. A day before testing mice were submitted to a habituation session, during which they were allowed to explore the empty apparatus for 5 min. Next day, the experimental session comprised two trials. In the first trial (T1), one object-stimulus, a sample (A), was placed near the rear wall of the box. During the second trial (T2), a new object (B) was added. The object (A′) presented during T2 was a duplicate of the sample presented in T1 (A) in order to avoid olfactory traits. Both objects, a new and a duplicate of the sample presented during T1, were placed on the opposite back corners. The objects to be discriminated between were made of glass and porcelain, and they differed in shape, color and size, which allowed recognizing them as novelty. Their weights were such that they could not be moved by animals. Positions and roles of objects (sample *vs* new object) were counterbalanced within each session. Moreover, the object had no natural significance for mice and had never been associated with reinforcement. Different pairs of objects were used for each session. The duration of T1 and T2 was 5 and 3 min, respectively. T2 started 60 min. after T1 began. An exploration was defined as the animal facing the object, with its nose within 2 cm from the object. Touching the object with nose and turning around was not considered as an exploration. Time spent by mice in objects’ exploration during T1 and T2 trials was measured manually using stopwatch. From this measure, the following variables were defined: A = exploration time of the sample presented during T1, B = exploration time of a new object presented during T2, (B + A′) = exploration time of a duplicate (A′) of the familiar object A and a new object (B) presented during T2. Object recognition was measured by index of discrimination (B − A′). Since (B − A′) may be biased by differences in overall levels of exploration, the discrimination ratio (B − A)/(B + A′) was also calculated. The latter one may vary between +1 and − 1. A positive score indicates more time spent with the novel object, a negative score indicates more time spent with the familiar object. A zero score indicates a null preference for objects (Aubele et al. 2008; Ennaceur et al. 1997). Moreover, the recognition index (RI) was calculated for each animal and expressed as a ratio: (B × 100)/(B + A′). The recognition index around 50% indicates that animal did not remember the familiar object (since time A and time B are comparable), while recognition index above 50% indicates, that animal has remembered the familiar object, since time B is longer than time A (Balderas et al. 2008; Ennaceur and Delacour 1988).

## Statistics

Statistical analyses were performed using Statistica 13.0 and GraphPad Prism 5. All data were first assessed for normality using the Shapiro–Wilk test. In the present study, some variables measured in object recognition test, and some evaluated in a holeboard test, as well as in elevated plus maze did not have normal distribution. Therefore, data from object recognition test, holeboard and elevated plus maze tests, were analyzed by one-way analysis of variance (ANOVA) with Bonferroni post hoc test or by Kruskal–Wallis followed with Dunn’s multiple comparison post hoc test, when appropriate. The effects of genotype and age on parameters measured in the object recognition test, holeboard and the in elevated plus maze were analyzed by General Linear Model (GLM). Differences were considered significant at *p* < 0.05. F values for ANOVA and GLM, as well as H values for Kruskal–Wallis test, degrees of freedom and *p* values were given only for significant differences. Due to the lack of interest in a sample presented during T1 trial of object recognition test one 4-month-old IL-6KO mouse was excluded from statistical analysis, even though it performed well in other behavioral tests, the holeboard and the elevated plus maze.

## Results

### Holeboard test

**Fig. 1:**
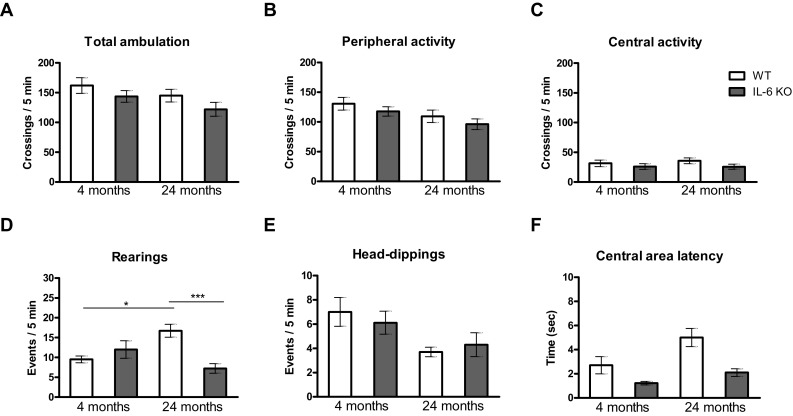
Effect of IL-6 deficiency on locomotor activity measured by **a** total, **b** peripheral and **c** central ambulation, and on exploratory activity measured by **d** rearings and **e** head-dips in a holeboard test performed on 4- and 24-month-old IL-6KO and age-matched WT mice. **f** Central area leaving time was used for the evaluation of anxiety level. Columns represent mean ± SEM of the values obtained from 9–10 animals. Rearings were less frequent in 24-month-old IL-6KO mice (***p < 0.005) and in 4-month-old WT ones (*p < 0.05) in comparison with 24-month-old WT animals (ANOVA and post hoc Bonferroni test)

There were no significant differences between tested groups in total ambulation (Fig. 1a), as well as in peripheral activity (Fig. 1b) and central activity (Fig. 1c) measured by crossings of squares adjacent and squares not adjacent to the apparatus walls, respectively. Out of two parameters used for the evaluation of exploratory activity: rearings (Fig. 1d) and head-dips (Fig. 1e) only rearing events differed between tested groups. ANOVA of rearings yielded F(3,35) = 7.330, *p* = 0.0006, and Bonferroni post hoc test revealed significant increase in incidences of rearings in 24-month-old WT animals in comparison with 24-month-old IL-6KO ones, *p* < 0.005, and to 4-month-old WT mice, *p* < 0.05 (Fig. 1d). Moreover, there were no significant differences between tested groups in latency to leave the central area, the parameter reflecting the level of anxiety (Fig. 1f).

Statistical analysis of genotype and age effects on parameters assessed in the holeboard by GLM revealed that peripheral activity and head-dips were age-dependent, rearings were genotype-dependent, while central area latency time was both age- and genotype-dependent (Supplementary Table I). GLM for peripheral activity yielded F(1,35) = 4.856, *p* < 0.05, and for head-dips yielded F(1,35) = 7.588, *p* < 0.01 indicating significant decrease of both parameters in 24-month-old vs 4-month-old mice. GLM for rearings yielded F(1,35) = 5.279, *p* < 0.05 indicating that rearing events were less frequent in IL-6KO mice that in WT mice. Regarding central area latency GLM yielded F(1,35) = 15.595, *p* < 0.01 for genotype and F(1,35) = 8.356, *p* < 0.01 for age showing that IL-6KO mice spent shorter and 24-month-old mice spent longer time in central area of the apparatus. Significant genotype x age interaction was observed only for rearings F(1,35) = 15.51, *p *< 0.005.

### Elevated plus maze

**Fig. 2:**
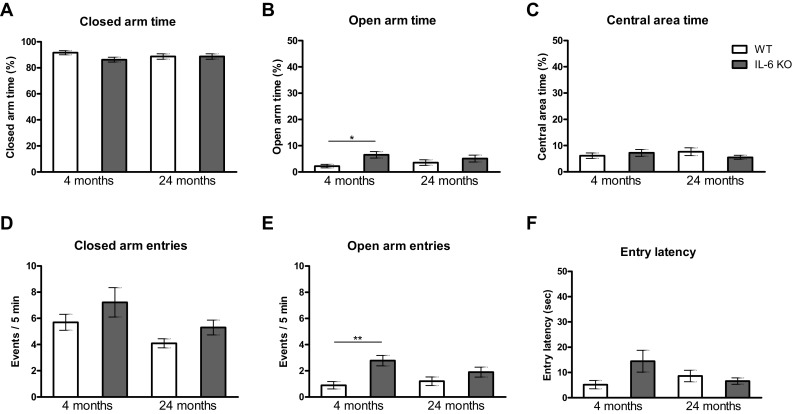
Effect of IL-6 deficiency on anxiety level evaluated in an elevated plus maze performed on 4- and 24-month-old IL-6KO and age-matched WT mice. Columns represent mean ± SEM of the values obtained from 9–10 animals. Percent of time spent **a** in closed arms, and **c** in central area of the apparatus, **f** central area latency time and number of entries **d** to closed arms were comparable in all tested groups. **b** 4-month-old IL-6KO mice spent significantly longer time (*p < 0.05) and **e** more frequently visited open arms (**p < 0.01) than 4-month-old WT animals (Kruskal–Wallis test followed by post hoc Dunn’s test)

Elevated plus maze test, assessing the level of anxiety, was performed immediately after the holeboard test. No significant differences were observed between 24-month-old IL-6KO and age-matched WT mice in all tested parameters: closed arm time (Fig. 2a), open arm time (Fig. 2b), and central area time (Fig. 2c), as well as in closed arm entries (Fig. 2d), open arm entries (Fig. 2e), and entry latency (Fig. 2f). Significant differences were observed between 4-month-old IL-6KO and age-matched WT mice only in open arm time (Fig. 2b) and in open arm entries (Fig. 2e). Kruskal–Wallis test yielded H(4,39) = 7.846, *p* = 0.0493 for open arm time and H(4,39) = 11.96, *p* = 0.0075 for open arm entries. Post-hoc comparison with Dunn’s test revealed significantly prolonged open arm time (*p* < 0.05) and significantly more entries to open arms (*p* < 0.01) in 4-month-old IL-6KO than in age-matched WT mice (Fig. 2b, e respectively).

When genotype and age effects on parameters assessed in the elevated plus maze were evaluated by GLM, significant increase in open arm time and significant increase in open arm entries were observed in IL-6KO mice in comparison with WT animals (Supplementary Table II). GLM yielded F(1,35) = 7.244, *p* < 0.05 for open arm time, and F(1,35) = 13.812, *p* < 0.01 for open arm entries. Moreover, aged animals significantly less often visited closed arms in comparison with young adult ones. GLM yielded F(1,35) = 6.414, *p* < 0.05. Significant genotype x age interaction was observed only for entry latency time F(1,35) = 4.936, *p *< 0.05.

### Object recognition test

**Fig. 3:**
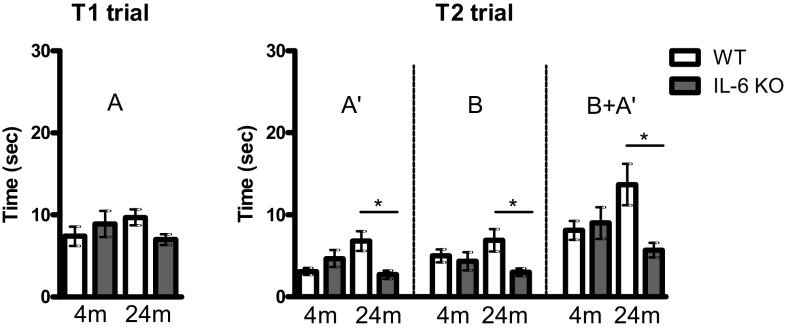
Effect of IL-6 deficiency on object’s exploration during T1 and T2 trials in an object recognition test performed on 4- and 24-month-old IL-6KO and age-matched WT mice. Columns represent mean ± SEM of the values obtained from 9–10 animals. **A**—exploration time of an object presented during T1 trial, **A′**—exploration time of a duplicate of familiar object presented during T2 trial, **B**—exploration time of a novel object presented during T2 trial, (**B + A′**)—the sum of exploration time of both objects presented in T2 trial. 24-month-old IL-6KO mice displayed shorter exploration of object B (ANOVA and post hoc Bonferroni test) and of object A′, as well as of both objects (B + A′) evaluated by Kruskal–Wallis test with Dunn’s post hoc test (*p < 0.05)

Time spent by mice on exploration of object A in T1 trial was comparable in four tested groups (Fig. 3). While the exploration time of a duplicate (A′) of the familiar object and a new object B during T2 trial were comparable in 4-month-old IL-6KO and WT mice, the time spent on A′, B and both objects’ (B + A′) exploration in 24-month-old mice were different (Fig. 3). Kruskal–Wallis test yielded H(4,39) = 10.79, *p* = 0.029 and H(4,39) = 9.462, *p* = 0.0237 for the time of objects’ A′ and B + A′ exploration, respectively, and further post hoc comparison with Dunn’s test revealed significantly shorter exploration (*p* < 0.05) of these objects by 24-month-old IL-6KO than age-matched WT mice. ANOVA for the time of object B exploration yielded F(3,35) = 2.814, *p* = 0.0354 and Bonferroni post hoc test showed significantly shorter time of object B exploration by 24-month-old IL-6KO than WT mice (*p* < 0.05).

**Fig. 4:**
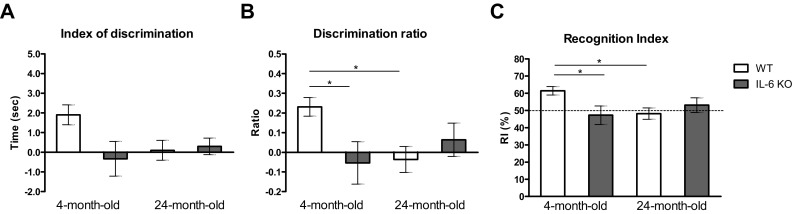
Effect of IL-6 deficiency on **a** index of discrimination (B − A′), **b** discrimination ratio (B − A′)/(B + A′) and **c** recognition index (B x 100)/(B + A′) evaluated in an object recognition test performed on 4- and 24-month-old IL-6KO mice and age-matched WT ones. Columns represent mean ± SEM of the values obtained from 9-10 animals. Discrimination ratios and recognition indexes were significantly lower in 4-month-old IL-6KO and in 24-month-old WT mice in comparison with 4-month-old WT animals (*p < 0.05, Kruskal–Wallis test with Dunn’s post hoc test)

Object recognition memory measured by the index of discrimination (difference B − A′) varied between 4-month-old WT mice and other tested groups but the differences were insignificant (Fig. 4a). Analysis of discrimination ratio (B − A′)/(B + A′) revealed significant attenuation of object recognition memory in 4-month-old IL-6KO mice and in 24-month-old WT mice in comparison with 4-month-old WT animals. Kruskal–Wallis test yielded H(4,39) = 10.79, *p* = 0.029, and further post hoc comparison with Dunn’s test revealed the same level of statistical significance for both groups (*p* < 0.05, Fig. 4b). Also, recognition index (B × 100/B + A′) was different between tested groups (Fig. 4c). While recognition index was over 60% in young adult WT mice indicating that after 1-hour delay they remembered familiar object, it was below 50% in 4-month-old IL-6KO and in 24-month-old WT animals indicating that the familiar object was not remembered. Kruskal–Wallis test yielded H(4,39) = 10.79, *p* = 0.029, and further post hoc comparison with Dunn’s test revealed significantly lower recognition index in both 4-month-old IL-6KO mice and in 24-month-old WT mice (*p* < 0.05). In 24-month-old IL-6KO mice the value of discrimination ratio reached 0.1 and recognition index exceeded 50% (53.17%), however they did not show significant differences with any of other tested groups.

GLM evaluation of genotype and age influence on parameters assessed in the object recognition test revealed that only exploration time of object B and of both objects (B + A′) during T2 trial was significantly shorter in IL-6-deficient than WT mice (*p* < 0.05), (Supplementary Table III). GLM for exploration time of object B yielded F(1,35) = 5.431, *p* < 0.05, and for exploration time of both objects (B + A′) F(1,35) = 4.138, *p* < 0.05. Significant genotype x age interactions were observed for all parameters except variables A and B (*p* < 0.05).

## Discussion

Results of the present study supported our previous observation that inborn IL-6 deficiency attenuates recognition memory in young adult mice in comparison with age-matched WT animals (Hryniewicz et al. 2007). The comparison of young adult with aged animals of both genotypes performed in the current study showed attenuation of recognition memory in 24-month-old WT animals as compared to 4-month-old WT ones, and lack of significant differences between 24-month-old IL-6KO mice and other tested groups. After 1-h delay both 4-month-old IL-6KO mice and 24-month-old WT animals did not differentiate a new from the familiar object because their recognition indexes were below 50% and their discrimination ratios had negative values, and these parameters were significantly lower than in 4-month-old WT animals. Although, in 24-month-old IL-6KO mice recognition index exceeded 50% (53.17%) it was not significantly different in comparison with all other groups. Moreover, while recognition index was significantly attenuated in aged vs young WT mice, in aged IL-6KO animals it was slightly higher than in young adult IL-6KO ones and reached 53.17%, indicating that their recognition memory was not worsened with age. However, it is impossible to exclude from the effect observed in aged IL-6KO mice the involvement of negative influence of inborn IL-6 deficiency on brain development that resulted in the attenuation of recognition memory observed in young adult IL-6KO animals. Although under physiological conditions IL-6 expression in brain tissue is very low, there is an evidence that IL-6 stimulates adult neurogenesis, possesses neurotrophic properties, regulates neuronal survival and function, and modulates neurotransmission in brain structures associated with cognitive processes (Balschun et al. 2004; D’Arcangelo et al. 2000; Erta et al. 2012; Gruol 2015; Islam et al. 2009; Kushima et al. 1992; Kushima and Hatanaka 1992). Moreover, IL-6 was proved to play a role in CNS development. Despite partial redundancy among different IL-6-type family members (Du et al. 1996; Ezure et al. 2000; Spooren et al. 2011) a disturbance of IL-6 signaling in the knock-out mice is likely to affect brain development, and as a consequence, neuronal functions correlated with memory processes, which may explain significant attenuation of recognition memory observed in 4-month-old IL-6KO mice.

Significant attenuation of recognition memory was reported also in 6-month-old IL-6KO mice (Baier et al. 2009), although experiments were performed in animals’ home cages and in the first session (T1 trial of our study) two objects were presented. Despite some procedural differences the recognition index in WT and IL-6KO mice (64% vs 49%) was similar to that in 12-14-week-old mice in our previous study (64.24% vs 50%) and also in 4-month-old mice in the current study (61.46% vs 48.18%). While Baier et al. (2009) concluded that observed effect could be partly explained by significantly reduced exploration time in the familiarisation session, in our study the time of object exploration during T1 trial was comparable in all tested groups. Moreover, reported in our study effect was memory-specific because IL-6KO mice performed more entries into the open arms of elevated plus maze and spent there longer time than WT animals. This indicates on their lower anxiety level, and that shorter exploration of the novel object in T2 trial in object recognition test was not dependent on neophobia. Also, there were no differences between genotypes in the holeboard test except shorter, although not statistically significant, central area latency time in IL-6-deficient mice supporting their lower anxiety level. Significantly higher number of rearings in 24-month-old WT than IL-6KO mice may explain significantly longer exploration of both, a duplicate of the familiar object A′, and the new object B in the T2 trial in the object recognition test.

In a study concerning cognitive processes in aged (24-month-old) IL-6-deficient and WT mice reported by (Dugan et al. 2009) short-term spatial recognition memory was tested by analysing habituation to the spatial distribution of objects in an open field. Aged IL-6- deficient mice showed a greater degree of habituation than age-matched WT animals indicating that they better remembered presented objects. However, in this experimental paradigm, but not in object recognition test used in our study, spatial component of recognition memory, dependent on hippocampus, was important. Therefore, while in our experiment pure visual-perceptual recognition memory was not worsened in aged vs young adult IL-6 KO mice, in Dugan et al. study (2009), performed in experimental paradigm with strong spatial component, IL-6 deficiency even protected against age-related recognition memory decline. Moreover, although in our previous study performed in Morris water maze learning ability (spatial working memory) was attenuated in both young adult and aged IL-6KO mice in comparison with age-matched WT ones (Bialuk et al. 2018), better retrieval process (spatial reference memory) in IL-6-deficient mice was observed (Bialuk et al. 2018; Bialuk and Winnicka 2018). This effect was probably caused by a different delay between learning and testing trial. In the object recognition test it was only 1 h, while in the Morris water maze between completion of 3-day learning and a single probe trial 24-h (Bialuk et al. 2018) or 7-day delay was introduced (Bialuk and Winnicka 2018). Balschun et al. (2004) reported that induced by learning LTP is followed by IL-6 expression detected 8 h later, which served as negative regulator of LTP, responsible for its termination. Therefore, in the experimental paradigms with long delay between learning and testing trial lack of IL-6 results in longer consolidation process mirrored by the improvement of retrieval process (reference memory). The putative mechanisms underlying different effect of IL-6 deficiency on spatial and recognition memory was described in details in our previous study (Bialuk and Winnicka 2018). Presented above behavioural studies, based on different experimental paradigms in which particular stages of learning and memory are evaluated, indicate that the involvement of IL-6 in cognitive processes is complex and multidirectional.

Accumulating evidence indicates that a crucial role in recognition memory plays perirhinal cortex (Brown and Aggleton 2001; Olarte-Sanchez et al. 2015; Winters et al. 2008; Winters and Bussey 2005), nevertheless, the involvement of hippocampus in some aspects of object recognition task performance was considered in numerous studies (Aggleton and Brown, 1999; Broadbent et al. 2004; Cassaday and Rawlins 1997; Rossato et al. 2007; Zola et al. 2000). Evidence from animal studies indicates that a system connected with perirhinal cortex is associated with discrimination of the familiarity and recency, whereas the hippocampus is associated with judging the prior occurrence of stimuli constellations. However, it has been also demonstrated that under rigorous testing conditions, an intact hippocampus is not essential for the judgement of the prior occurrence of an object (Winters et al. 2004). Moreover, the findings reported by (Winters and Bussey 2005) supported a role for perirhinal cortex neuronal activity in encoding, consolidation and retrieval of the object recognition memory. In support of this hypothesis, electrophysiological recording studies have provided evidence of neuronal changes related to familiarity of a visual stimulus in the anterior temporal lobe cortex (entorhinal and perirhinal cortices) (Brown et al. 1987; Miller et al. 1991). The involvement of IL-6 in age-related memory disturbances was demonstrated in senescence-accelerated mice (Tha et al. 2000) and in clinical observations on humans. Significant increase of IL-6 expression measured by ELISA in cerebral cortex and in hippocampus was also shown in 24-month-old BALB/c mice in comparison with 1- and 3-month-old animals (Ye and Johnson 1999). However, molecular mechanisms by which IL-6 overexpression lead to cognitive impairment have not been fully elucidated. In postmortem study performed in patients with severe dementia significantly elevated mRNA for IL-6 and TGF-β1 levels in the entorhinal cortex as compared with cognitively normal subjects was reported, whereas IL-1β mRNA was very low (Luterman et al. 2000). Massey et al. (Massey et al. 2001) demonstrated that a cholinergic mechanism of synaptic plasticity within perirhinal cortex may play a role in different aspects of perirhinal-mediated object recognition memory processes. It has been shown that choline acetyltransferase activity was significantly lower in IL-6KO than in control mice (Braida et al. 2004), and that elevated level of IL-6 disrupts cholinergic transmission by altering metabotropic glutamate receptor-activated calcium signalling (Nelson et al. 2004). Therefore, both significant decrease of acetylcholine in IL-6KO mice, as well as disruption of cholinergic transmission by increased IL-6 level may be responsible for attenuation of recognition memory observed in IL-6-deficient and in 24-month-old WT animals with age-related elevation of IL-6 concentration, respectively.

Expression of IL-6 increases with age and experiments performed on animals with overexpression of IL-6 showed that not only IL-6 deficiency but also high level of this cytokine affected mechanism of neuroplasticity underlaying memory processes (Bellinger et al. 1995; Nelson et al. 2012; Steffensen et al. 1994), and can interfere with adult neurogenesis thus contributes to the impairment of cognitive functions (Vallieres et al. 2002). Epidemiological studies have shown that peripheral IL-6 levels were inversely correlated with cognitive functions in aged subjects (Ershler and Keller 2000; Sarkisian et al. 2008) and chronic peripheral elevation of this cytokine was associated with mild cognitive deficits even in apparently healthy older adults (Bermejo et al. 2008; Marsland et al. 2006). Furthermore, elevated IL-6 level constitutes a significant predictor of transition from mild cognitive impairment to Alzheimer’s disease (Bermejo et al. 2008; Ershler and Keller 2000) and progression of this disease in older individuals (Ershler and Keller 2000; Maggio et al. 2006).

Present study supported our previous finding that inborn IL-6 deficiency attenuated recognition memory in young adult mice, and demonstrated significant attenuation of recognition memory in aged WT animals, and that the latter effect was not altered by IL-6 deficiency. However, in experiments performed on aging IL-6KO animals it is impossible to rule out the negative effect of IL-6 deficiency on CNS development. Taking into consideration that inborn IL-6 deficiency impairs CNS development, similar inability of discrimination between a new and the familiar object observed in aged IL-6KO and WT animals reported in the present study does not exclude the possibility, that increasing with age IL-6 expression and especially its overexpression, may impact age-related memory decline. Therefore, IL-6 signalling may constitute a target for development of the protection against memory disturbances connected with IL-6 overexpression.

## Electronic supplementary material

Below is the link to the electronic supplementary material.

Supplementary material 1 (PDF 100 kb)
