# Supplementary material for: Significance of IL-6 Deficiency in Recognition Memory in Young Adult and Aged Mice
Source: Behav Genet. 2019 May 25;49(4):415–23. doi: 10.1007/s10519-019-09959-6 (PMC6554246; doi:10.1007/s10519-019-09959-6)
Supplement: Supplementary file 1 — Supplementary material 1 (PDF 100 kb) [file 10519_2019_9959_MOESM1_ESM.pdf]

Supplementary material

Supplementary Table I. Results of holeboard test performed a day before object recognition test with respect to genotype and age of mice.

Variables	**WT**	**IL-6KO**	**4-month-old**	**24-month-old**
	N = 20	N = 19	N = 19	N = 20
**Locomotor activity measured by crossings**				
Total ambulation	153.500±8.465	132.300±7.929	153.300±8.395	133.500±8.144
Peripheral activity	120.000±7.672	106.400±6.354	124.500±6.776	102.900±6.827*
Central activity	33.500±3.602	25.840±3.147	28.840±3.659	30.650±3.355
**Exploratory activity measured by number of events**				
Rearings	13.100±1.218	9.474±1.319*	10.680±1.142	11.950±1.473
Head-dips	5.350±0.715	5.158±0.702	6.579±0.754	4.000±0.523**
**Central area latency**				
Time (sec)	3.850±0.567	1.684±0.203**	2.000±0.411	3.550±0.515**

Table presents means ± SEM of the values obtained from 19-20 animals. According to GLM peripheral activity was significantly lower in 24-month-old mice vs. 4-month-old animals, * $p < 0.05$ and it was not influenced by genotype. Total ambulation and central activity were neither influenced by genotype nor by age. Rearings were less frequent in IL-6KO mice while head-dipping events in 24-month-old animals. Central area leaving time, used for the evaluation of anxiety level, was significantly shorted in IL-6KO mice and longer in 24-month-old ones, ** $p < 0.01$.

Supplementary Table II. Results of elevated plus maze performed a day before object recognition test with respect to genotype and age of mice.

Variables	**WT**	**IL-6KO**	**4-month-old**	**24-month-old**
	N = 20	N = 19	N = 19	N = 20
**% of time spent in**				
Closed arms	90.170 ± 1.319	87.540 ± 1.404	89.050 ± 1.348	88.730 ± 1.433
Open arms	2.900 ± 0.626	5.789 ± 0.893*	4.281 ± 0.831	4.333 ± 0.838
Central area	6.933 ± 0.899	6.333 ± 0.735	6.667 ± 0.795	6.617 ± 0.857
**Number of entries into**				
Closed arms	4.900 ± 0.390	6.211 ± 0.633	6.421 ± 0.632	4.700 ± 0.348*
Open arms	1.500 ± 0.211	2.316 ± 0.286**	1.789 ± 0.320	1.550 ± 0.256
**Entry latency**				
Time (sec)	6.900 ± 1.608	10.320 ± 2.279	9.579 ± 2.410	7.600 ± 1.276

Table presents means ± SEM of the values obtained from 19-20 animals. Both, open arm time and open arm entries were genotype-dependent. GLM revealed that IL-6KO mice entered more often open arms (**$p < 0.01$) and spent there longer time (* $p < 0.05$). Closed arm entries were less frequent in 24-month-old mice than in 4-month-old mice, * $p < 0.05$. Closed arm time, central area time, as well as entry latency were genotype- and age-independent.

Supplementary Table III. Results of object recognition test with respect to genotype and age of mice.

Variables	**WT**	**IL-6KO**	**4-month-old**	**24-month-old**
	N = 20	N = 19	N = 19	N = 20
**Exploration (sec)**				
A	8.550 ± 0.793	7.895 ± 0.840	8.105 ± 0.973	8.350 ± 0.650
B	5.950 ± 0.792	3.632 ± 0.578*	4.684 ± 0.649	4.950 ± 0.828
A’	4.950 ± 0.755	3.642 ± 0.593	3.842 ± 0.550	4.750 ± 0.794
B+A’	10.900 ± 1.495	7.263 ± 1.076*	8.526 ± 1.080	9.700 ± 1.591
**Discrimination**				
B-A’ (sec)	1.000 ± 0.403	0.000 ± 0.465	0.841 ± 0.547	0.200 ± 0.321
B-A’/B+A’ (sec)	0.097 ± 0.050	0.007 ± 0.067	0.095 ± 0.064	0.013 ± 0.535
(B×100)/(B+A′) (%)	54.820 ± 2.512	50.380 ± 3.373	54.750 ± 3.242	50.670 ± 2.677

Table presents means ± SEM of the values obtained from 19-20 animals. A = exploration time of the sample during T1 trial; B = exploration time of the new object during T2 trial; A’ = exploration time of a duplicate of the familiar object A during T2 trial; (B+A') = exploration time of a duplicate of the familiar object A (A') and a new object (B) during T2; (B-A’) = index of discrimination, (B-A’/B+A’) = discrimination ratio, (B×100)/(B+A′) = recognition index. Statistical analysis with GLM showed significant differences only for variables B and B+A’, that were both genotype-dependent. IL-6KO mice spent shorter time on exploration of object B in T2 trial in comparison with WT mice, * $p < 0.05$. Also, sum of exploration time of both objects (B+A’) presented in T2 trial was shorter in IL-6KO than in WT mice, * $p < 0.05$. Significant genotype x age interactions were observed for all parameters at the $p < 0.05$, except variables A and B.
